# Can the Severity of Normal Tissue Damage after Radiation Therapy Be Predicted?

**DOI:** 10.1371/journal.pmed.0030440

**Published:** 2006-10-31

**Authors:** Adrian C Begg

## Abstract

Begg discusses a new study by Svensson and colleagues in which the researchers attempted to elucidate genetic factors involved in late radiation toxicity.

Radiotherapy can lead to both acute and late side effects, the former occurring over a timescale of weeks and then subsiding. Late effects occur months or even years after cessation of treatment and often show continuous progression with time. With conventionally fractionated radiotherapy, the late side effects usually limit the dose.

There are clear differences between patients regarding side effects: some patients appear to tolerate the treatment well, while others develop severe symptoms. Radiotherapy dose schedules have therefore been designed on past experience so that no more than 5%–10% of patients develop such severe side effects.

The question that has occupied radiation oncologists and scientists for many years is: what is different about the patients who develop the severe side effects? If we knew this, these patients could be given alternative schedules, doses, or treatments in order to produce more tolerable effects. In addition, and perhaps more importantly, the remaining majority of patients could be given a higher dose, leading to higher cure rates [1]. The question is therefore potentially important, but has not been an easy one to answer.

## Can Lymphocyte Response Predict Patient Morbidity?

In a new study published in *PLoS Medicine*, J. Peter Svensson and colleagues present an intriguing attempt to elucidate genetic factors involved in late radiation toxicity [2]. Their approach was to look at differences in gene expression in lymphocytes of patients treated for prostate cancer. They hoped to discriminate patients with severe late radiation complications following radiotherapy (“over-responders” [OR]) from patients without such complications (“non-responders” [NR]). The OR group showed severe late complications of rectum and/or

The question is: what is different about the patients who develop severe side effects?

bladder, tissues which are unavoidably included in the radiation field. Twenty-one over-responders and 17 non-responders participated in the primary classification study. Twelve patients (6 ORs and 6 NRs) were used for independent validation.

Peripheral blood lymphocytes from both the NR and OR groups were stimulated to proliferate with phytohaemagglutinin for two days. The lymphocytes were irradiated with 2 Gy (the standard daily radiotherapy dose) and then RNA was extracted 24 hours later for microarray analysis. Changes in gene expression resulting from the ex vivo irradiation were found to correlate with OR and NR status, although the performance of the classifier was only moderate (the classifier based on the radiation response of separate genes correctly classified 63% of the patients). Better performance was achieved by considering sets of genes on specific functional pathways based on the Gene Ontology categories, including those for apoptosis, protein metabolism and ubiquitination, development, and stress signaling. Such gene sets were able to predict OR and NR status with an 83% accuracy. If validated, this would represent a step forward for the radiation oncologist.

## Strengths and Weaknesses of the Study

There are two major strengths of this study. The first is the selection of a good number of over-responders (27) from an initially large series (800) of patients treated in a single institute for prostate cancer. Identifying and then collecting material for analysis from this relatively homogeneously treated group is a valuable achievement. Second, the analysis of sets of functionally related genes, in addition to the usual approach of treating each gene separately, was clearly a step in the right direction, a trend now seen in many microarray studies.

Paradoxically, the number of patients in the study is also a weakness. The number of events (serious side effects) eventually found and used for the training series was not large (21) and the validation population was small (6 ORs + 6 NRs). This was reflected in a moderate performance on the validation group, eight of 12 being correctly predicted, not significantly different from random chance. As admitted by the authors, part of this poor performance may have been due to slight differences in the handling of lymphocytes. Further studies are therefore required to see if these intriguing, preliminary results hold up. As with all microarray studies, finding a classifier in a training group is not difficult. Finding a robust predictor withstanding validation in independent trials has turned out to be a lot more challenging.

## Why Lymphocytes?

If these results do hold up after further investigation, another question arises. The normal tissues damaged by radiation in this study were bladder and rectum. Some dose to these organs cannot be avoided, even by modern conformal radiotherapy. The question is: why should gene expression in a lymphocyte predict what will happen in these different and complex tissues, comprising largely epithelial, stromal, and vascular cells? The assumption is that there are underlying genetic factors governing the response of most or all tissues in the body to radiation. This is not immediately obvious. Radiation pathogenesis in such tissues depends on a number of factors, including damage to parenchymal cells and vasculature, and often involves various cytokines (e.g., TGF-beta) [3]. These factors will not be involved, or will only be involved to a lesser extent, in the response of lymphocytes.

In defense of Svensson and colleagues' approach, at least two studies have shown that the extent of cytogenetic damage in lymphocytes irradiated ex vivo correlates with normal tissue damage after radiotherapy [4,5]. Correlations of cytogenetic damage with gene expression would then support the present approach. There is little information on this, although it is logical to assume that intrinsic radiosensitivity differences will be reflected in expression or function of the many genes affecting radiosensitivity. Reiger and colleagues [6] showed that expression changes in lymphoblastoid lines derived from patients and irradiated ex vivo correlated with radiation-induced morbidity, although again the study was small (14 patients).

## Questions and Future Directions

Tissue and vascular factors undoubtedly influence the pathogenesis of normal tissue damage, and can vary between patients. Lymphocyte expression studies will not address these factors. If lymphocyte gene expression indeed turns out to be highly predictive in further investigations, it would imply that such tissue-related factors play only a minor role, which would represent a surprising and interesting finding.

More probably, tissue factors, maybe organ specific, will be found to play a role, and will eventually also need to be taken into account in any predictive test. Assuming genetic factors determine normal tissue damage, these could indeed be analyzed in lymphocytes, but at the DNA level. Single nucleotide polymorphisms, for example, have already shown some promise in predicting normal tissue morbidity [7]. Using lymphocytes for this purpose may be more fruitful than analyzing gene expression, but time will tell. The statistical power of Svensson and colleagues' study is too low to warrant unbridled optimism at present, although it will surely stimulate further investigations and hopefully lead to improvements in radiotherapy.

## Abbreviations

NR: non-responder
OR: over-responder
